# Comparative *in vitro* antimicrobial susceptibility profiles of clofazimine and pyrifazimine against clinical isolates of *Mycobacterium tuberculosis* in southwest China

**DOI:** 10.1128/spectrum.02664-25

**Published:** 2026-07-06

**Authors:** Ming Luo, Gang Zhou, Yunfan Fan, Min Dai, Yunusijiang Wubulikasimu, Chuanning Tang, Feifei Wang, Pengsen Wang, Lele Wang, Qian Cheng, Peibo Li

**Affiliations:** 1 Clinical Laboratory, Chongqing Public Health Medical Center 567508, Chongqing, China; 2 Tuberculosis Department, Chongqing Public Health Medical Center 567508, Chongqing, China; 3 Clinical Laboratory, Shufu County People’s Hospital, Xinjiang, China; 4 Key Laboratory of Tropical Translational Medicine of Ministry of Education, Hainan Medical University-The University of Hong Kong Joint Laboratory of Tropical Infectious Diseases, Hainan Medical University 12455, Haikou, China; 5 Administration Office of the Hospital, Chongqing Public Health Medical Center 567508, Chongqing, China; 6 Clinical Research Center, Chongqing Public Health Medical Center 567508, Chongqing, China; Montefiore Medical Center and Albert Einstein College of Medicine, Bronx, New York, USA

**Keywords:** clofazimine, pyrifazimine, drug resistance, antimicrobial susceptibility, mutation analysis, *Mycobacterium tuberculosis*

## Abstract

Clofazimine (CFZ) is a key drug used to treat drug-resistant tuberculosis (DR-TB), while pyrifazimine (TBI-166) is an improved riminophenazine derivative with better pharmacokinetics. However, there is a lack of data on its susceptibility and resistance in regions with a high disease burden, such as southwestern China. We compared the *in vitro* antimicrobial activities of CFZ and TBI-166 against 249 DR-TB clinical isolates (99 multidrug-resistant TB [MDR-TB] and 150 pre-extensively drug-resistant TB [pre-XDR-TB] isolates) from southwestern China. TBI-166 exhibited a concentration-dependent biphasic antimicrobial pattern compared to CFZ. TBI-166 showed significantly greater potency at low concentrations (MIC₅₀ = 0.031 µg/mL TBI-166 vs 0.25 µg/mL for CFZ; *P* < 0.001), but attenuated inhibition at high concentrations (MIC₉₀ > 4 µg/mL vs 1 µg/mL for CFZ; *P* < 0.001). However, at high concentrations, the antibacterial effect of TBI-166 is weaker than that of CFZ (40% of TBI-166-resistant isolates have MIC > 4 µg/mL, compared to 6.67% of CFZ-resistant isolates, *P* < 0.001). Epidemiological cutoff values (ECOFFs) were 1.0 µg/mL for CFZ and 0.25 µg/mL for TBI-166. Based on these *in vitro* ECOFFs, the resistance rate to TBI-166 (20.1%, 50/249) was significantly higher than that to CFZ (6.0%, 15/249, *P* < 0.0001). Whole-genome sequencing revealed that mutations in *Rv0678* were prevalent in dual-resistant isolates (10/14) and TBI-166 monoresistant isolates (5/40), while *Rv1979c* mutations were less frequent, and no *pepQ* mutations were detected. These mutations differed from known hotspots, suggesting potential novel resistance mechanisms.

**IMPORTANCE:**

Drug-resistant tuberculosis (DR-TB) remains a major global health challenge, and optimizing treatments for high-burden regions like southwest China is crucial. This study is the first to detail the differential *in vitro* activities of clofazimine (CFZ) and the novel TBI-166 against clinical DR-TB isolates from southwest China, alongside their resistance-associated genetic profiles. Findings show TBI-166 has enhanced low-concentration potency but higher resistance rates, plus novel mutations in *Rv0678* and *Rv1979c* linked to resistance. These insights will help refine clinical regimens for DR-TB and strengthen regional resistance surveillance, both of which are essential for controlling the spread of DR-TB in southwest China and informing treatment and surveillance strategies in other similar high-burden areas globally.

## INTRODUCTION

Tuberculosis (TB) remains the leading cause of mortality globally from a single infectious pathogen. The 2023 World Health Organization (WHO) Global TB Report estimated 1.25 million TB-related deaths, exceeding HIV/AIDS-related fatalities by twofold, along with 10.8 million new cases, including 400,000 multidrug-resistant/rifampin-resistant TB (MDR/RR-TB) cases (1). As a high-burden country, China reported ~29,000 new MDR/RR-TB cases (7.3% of the global total), ranking fourth worldwide. Drug-resistant TB (DR-TB), particularly MDR-TB (resistance to both isoniazid and rifampin) and pre-extensively drug-resistant TB (pre-XDR-TB, defined as MDR-TB with additional resistance to any fluoroquinolone) (2), poses a critical threat, highlighting the need for improved antimicrobials. Notably, DR-TB genotypic profiles, including mutations associated with riminophenazine resistance, exhibit significant geographical variation. Southwest China, a high-endemicity region with substantial MDR/pre-XDR-TB burden (7.3% MDR-TB prevalence, consistent with global hotspots like Southeast Asia and sub-Saharan Africa) (1), lacks characterization of riminophenazine resistance genotypes, limiting localized therapeutic optimization. Critically, as a key transportation hub along the Belt and Road Initiative, it further underscores the relevance of its resistance profiles to cross-border transmission control.

CFZ, a repurposed antileprosy agent, is recommended as a key drug in short- and long-term individualized DR-TB regimens (3) and is under evaluation in trials for novel shorter regimens targeting both DR-TB and drug-susceptible TB (4, 5). In clinical practice, CFZ has shown promise in MDR-TB treatment. An observational study in Bangladesh reported an 87% success rate with 9–11-month CFZ-containing short regimens, compared to 50%–60% with conventional long-course injectable regimens for MDR/RR-TB (6). Preclinical studies further confirmed that CFZ shortens treatment duration when integrated into first- and second-line combination therapies (7). In 2019, CFZ was designated a Group B core drug for MDR-TB by both the WHO in its *Integrated Guidelines for the Treatment of Drug-Resistant Tuberculosis* (8) and the Chinese Anti-Tuberculosis Association in its *Chemotherapy Guidelines for Drug-Resistant Tuberculosis* (2019 Short Version) (9). Despite better tolerability than other second-line agents in DR-TB treatment, CFZ use in clinical settings is limited by adverse events (e.g., skin pigmentation, gastrointestinal disturbances) that compromise patient adherence.

Pyrifazimine (TBI-166), a novel riminophenazine derivative structurally modified from CFZ via C-3 pyridine ring adjustment to optimize lipid solubility, is currently in Phase II clinical trials (registration number: CTR20202345). Compared to CFZ, TBI-166 exhibits enhanced *in vitro* activity against drug-sensitive, drug-resistant, intracellular, and non-replicating *Mycobacterium tuberculosis*. Its favorable pharmacokinetic profiles, including lower lipophilicity (logP = 4.52) and a shorter half-life (t_1/2_ = 41.25 h) (10), support broader clinical potential. In murine models of acute and chronic low-dose aerosol-induced TB, TBI-166 activity was comparable to CFZ, with lung bacterial loads ~1% of CFZ-treated controls at equivalent doses (10). Additionally, despite higher tissue accumulation at equal doses, TBI-166 reduces skin pigmentation relative to CFZ across all time points (11). As a CFZ analog, TBI-166 may share partial resistance pathways (e.g., efflux pump-mediated mechanisms) due to their structural similarity. However, its modified lipid solubility and pharmacokinetic profiles suggest the existence of distinct resistance mutations that have not yet been characterized in clinical isolates.

The mechanism of CFZ activity against *M. tuberculosis* remains incompletely defined, but it is hypothesized to target cellular membranes, disrupting processes such as respiration and ion transport (12). Recent studies linked elevated CFZ MICs to mutations in the genes of *Rv0678*, *Rv1979c*, and *Rv2535c* (*pepQ*) (13–15). *Rv0678*, a regulatory factor, modulates expression of the MmpS5-MmpL5 efflux pump; mutations in this gene and upregulated *mmpl5* expression may drive cross-resistance between CFZ and bedaquiline (13). *Rv1979c*, encoding a membrane transporter with permease activity (putatively involved in CFZ translocation/uptake), confers increased CFZ MICs when mutated (16). PepQ (encoded by *Rv2535c*), a proline aminopeptidase, mediates low-level CFZ and bedaquiline resistance upon functional inactivation via mutation (15).

Despite the promise of TBI-166 as an alternative to CFZ, some critical gaps remain. These include regional variations in DR-TB genotypes, with MDR/pre-XDR-TB isolates from the southwestern Chinese region with a high disease burden remaining understudied; unclear relevance of characterized CFZ resistance genes (*Rv0678*, *Rv1979c*, *pepQ*) to structurally modified TBI-166; and a lack of direct *in vitro* activity comparisons in clinical isolates from endemic regions. Here, we compared the activity of CFZ and TBI-166 against MDR/pre-XDR-TB isolates from southwestern China, analyzed mutations in these genes to elucidate riminophenazine resistance mechanisms, and established epidemiological cut-off values (ECOFF) to inform clinical susceptibility interpretation, regional therapeutic optimization, and resistance surveillance.

## MATERIALS AND METHODS

### Clinical isolates and culture conditions

A total of 249 clinical DR-TB isolates were selected from consecutive patients admitted to Chongqing Public Health Medical Center (Chongqing, China) between October 2017 and October 2023. Isolates were included in this study based on successful recovery and viability from cryopreservation (rather than random sampling), with the annual distribution as follows: 2017 (*n* = 18), 2018 (*n* = 87), 2020 (*n* = 16), 2021 (*n* = 5), 2022 (*n* = 39), and 2023 (*n* = 84). The comprehensive information was gathered and documented for all patients, including age, gender, TB contact history, and TB treatment history. Each isolate was derived from a unique patient and classified as MDR-TB or pre-XDR-TB according to the pre-2023 WHO definition. Drug susceptibility was determined using the absolute concentration method on Löwenstein-Jensen (L-J) medium containing the corresponding anti-TB drugs following the WHO guidelines (17). Isolates were stored in 7H9 broth supplemented with 15% glycerol at −80°C. Before *in vitro* susceptibility testing, the isolates were recovered on L-J medium at 37°C for 4 weeks.

### Minimum inhibitory concentration testing determination

The microplate Alamar Blue Assay was used to determine the MICs of CFZ and TBI-166 against DR-TB isolates, as described previously (11, 17). Briefly, 100 µL of 7H9 broth containing serial dilutions of CFZ or TBI-166 was added to the 96-well plates, with the final concentration ranges 0.016 to 16 µg/mL (0.016, 0.031, 0.0623, 0.125, 0.25, 0.5, 1, 2, 4, 8, and 16 µg/mL) for CFZ and 0.004 to 4 µg/mL (0.004, 0.008, 0.016, 0.031, 0.0623, 0.125, 0.25, 0.5, 1, 2, and 4 µg/mL) for TBI-166. Drug-free wells served as blank controls. Bacterial colonies were harvested from the L-J slant surfaces, vortexed for 1 min, and adjusted to a 1.0 McFarland. The inoculum was diluted 1:20 in Middlebrook 7H9 broth supplemented with 10% oleic acid-albumin-dextrose-catalase, and 100 μL of this suspension was added to each well containing the corresponding drugs. After 7 days of incubation at 37°C, 70 μL of Alamar Blue solution (20 µL resazurin + 50 µL 5% Tween-80) was added, followed by a 24 h incubation at 37°C. Fluorescence was measured at 530 nm excitation and 590 nm emission wavelengths. The MIC was defined as the lowest drug concentration reducing fluorescence by ≥90% relative to the mean of drug-free controls. MTB H37Rv was used as a drug-susceptible control, and all samples were tested in duplicate to ensure reproducibility.

### Determination of epidemiological cutoff values

The ECOFF was determined by a combination of an intuitive method and a statistical method. For visual estimation, we first generated frequency histograms of MIC distributions for all clinical isolates. The ECOFF was initially identified as the MIC value marking the end of the wild-type (WT) distribution peak, which was typically 1–2 doubling dilutions above the highest MIC concentration where the WT population (susceptible isolates) clustered. For statistical validation, we used the ECOFFinder software (https://clsi.org/meetings/susceptibility-testing-subcommittees/ecoffinder/) recommended by the Clinical and Laboratory Standards Institute to simulate ECOFF values at 95%, 97.5%, 99%, 99.5%, and 99% confidence intervals. The final ECOFF for CFZ and TBI-166 was confirmed by reconciling results from both methods, ensuring consistency between visual patterns and statistical thresholds.

### Whole-genome sequencing

Whole-genome sequencing (WGS) was performed on pre-treatment clinical isolates as previously described (18). Briefly, bacterial colonies were harvested from the L-J slant surfaces and suspended in 500 μL Tris-EDTA buffer, followed by high-temperature inactivation at 80°C for 30 min. For DNA from *in vitro* evolution experiments, extraction was performed using the cetyltrimethylammonium bromide method, with sequencing conducted on the Illumina NextSeq500 platform (Uni-medica, Shenzhen, China), generating an average sequencing depth of ≥100× and ≥95% genome coverage of the MTB H37Rv reference genome (GenBank: NC_000962.3). DNA libraries were constructed using the TIANSeq Direct Fast DNA Library Prep Kit (Tiangen Biotech, Beijing, China; Catalog No. NG101-T2) according to the manufacturer’s protocols, and sequences were aligned to the *Mycobacterium tuberculosis* (MTB) H37Rv reference genome via the SAM-TB platform (19). Focused analysis was performed on CFZ and TBI-166 resistance genes in MTB, including *Rv0678*, *Rv1979c*, and *pepQ*. Additionally, studies indicate that *Rv2621c*, *Rv2622*, and *Rv3418c* are associated with TBI-166 resistance (20); these genes were exploratory candidates for preliminary screening, not part of a pre-defined core analysis.

### Statistical analysis

The Pearson chi-square or Fisher’s exact test was used to compare proportions or rates. Each violin plot presents the distribution and probability density of the data. Statistical analysis was performed using SPSS version 22.0 software (SPSS Inc., Chicago, IL). Differences were considered statistically significant for *P* < 0.05.

## RESULTS

### CFZ and TBI-166 MICs for MDR- and pre-XDR-TB isolates

**Fig 1:**
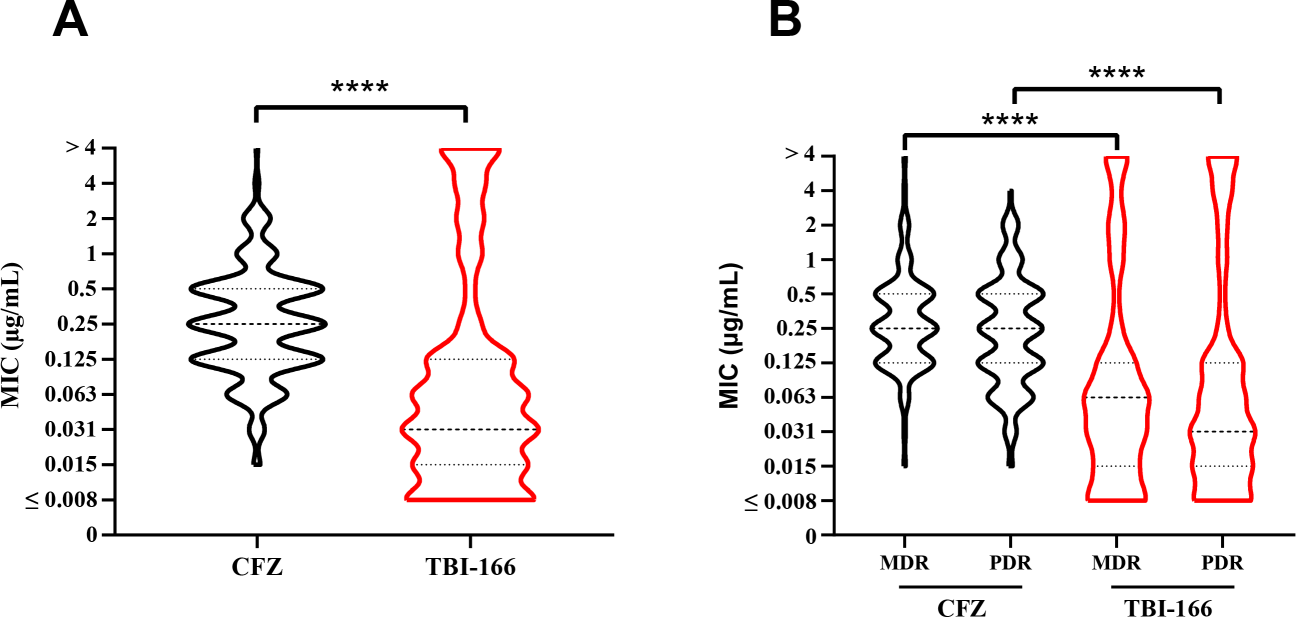
MIC distributions of CFZ and TBI-166 against MTB. (**A**) *In vitro* antibacterial activity of CFZ and TBI-166 against all DR-TB isolates; (**B**) MIC distributions stratified by MDR-TB and pre-XDR-TB subgroups. Data are presented as violin plots, where the width of each violin represents the kernel probability density of the MIC data (wider sections indicate a higher density of isolates at that MIC value). Different colored plots denote specific drugs: black for CFZ and red for TBI-166. Statistical differences were analyzed using nonparametric tests, followed by Dunnett’s multiple comparison test. ****P < 0.0001. MIC, minimum inhibitory concentration; MDR, multidrug-resistant *Mycobacterium tuberculosis*; PDR, pre-extensively resistant *Mycobacterium tuberculosis*.

The MIC distributions of CFZ and TBI-166 against 249 *M. tuberculosis* isolates (99 MDR-TB isolates and 150 pre-XDR-TB isolates) are presented in Table 1 and Fig. 1. Overall, TBI-166 exhibited a biphasic susceptibility profile, with a MIC₅₀ of 0.031 µg/mL (eightfold lower than CFZ’s MIC₅₀ of 0.25 µg/mL) and a MIC₉₀ of 4 µg/mL (fourfold higher than CFZ’s MIC₉₀ of 1 µg/mL). The median MIC of TBI-166 was 0.031 [0.015, 0.125] µg/mL, significantly lower than that of CFZ (0.25 [0.125, 0.5] µg/mL; *P* < 0.001).

**TABLE 1:** Distribution of *M. tuberculosis* isolates with different CFZ and TBI-166 MIC values^*a*^

Classification and drugs	No. (%) of isolates with MIC (µg/mL)												MIC_50_ (µg/mL)	MIC_90_ (µg/mL)
	≤0.008 (µg/mL)	0.015 (µg/mL)	0.031 (µg/mL)	0.063 (µg/mL)	0.125 (µg/mL)	0.25 (µg/mL)	0.5 (µg/mL)	1 (µg/mL)	2 (µg/mL)	4 (µg/mL)	>4 (µg/mL)	Total		
MDR-TB														
CFZ	0 (0.0)	1 (1.0)	1 (1.0)	4 (4.0)	26 (26.3)	29 (29.3)	26 (26.3)	6 (6.1)	4 (4.0)	1 (1.0)	1 (1.0)	99	0.25	0.5
TBI-166	18 (18.2)	12 (12.1)	17 (17.2)	19 (19.2)	11 (11.1)	3 (3.0)	1 (1.0)	4 (4.0)	5 (5.1)	2 (2.0)	7 (7.1)	99	0.063	2
Pre-XDR-TB														
CFZ	0 (0.0)	2 (1.3)	6 (4.0)	23 (15.3)	33 (22.0)	32 (21.3)	33 (22.0)	12 (8.0)	8 (5.3)	1 (0.7)	0 (0.0)	150	0.25	1
TBI-166	25 (16.7)	25 (16.7)	28 (18.7)	19 (12.7)	18 (12.0)	4 (2.7)	2 (1.3)	4 (2.7)	4 (2.7)	8 (5.3)	13 (8.7)	150	0.031	4
Total														
CFZ	0 (0.0)	3 (1.2)	7 (2.8)	27 (10.8)	59 (23.7)	61 (24.5)	59 (23.7)	18 (7.2)	12 (4.8)	2 (0.8)	1 (0.4)	249	0.25	1
TBI-166	43 (17.3)	37 (14.9)	45 (18.1)	38 (15.3)	29 (11.6)	7 (2.8)	3 (1.2)	8 (3.2)	9 (3.6)	10 (4.0)	20 (8.0)	249	0.031	4

^*a*^ MDR-TB, multidrug-resistant *Mycobacterium tuberculosis*; pre-XDR-TB, pre-extensively resistant *Mycobacterium tuberculosis*; MIC_50_, the minimum concentration of an antimicrobial agent required to inhibit the growth of 50% of the organisms; MIC_90_, the minimum concentration of an antimicrobial agent required to inhibit the growth of 90% of the organisms.

In subgroup analyses, TBI-166 exhibited lower MIC_50_ values than CFZ in both MDR-TB (0.063 vs 0.25 μg/mL) and pre-XDR-TB (0.031 vs 0.25 µg/mL) isolates. Conversely, TBI-166 displayed fourfold higher MIC_90_ values (2 μg/mL for MDR-TB and 4.0 μg/mL for pre-XDR-TB) than CFZ (0.5 μg/mL and 1.0 μg/mL, respectively). The MIC distributions of CFZ and TBI-166 differed significantly between MDR-TB and pre-XDR-TB groups (*P* < 0.0001), though no significant within-group differences in MIC values were observed for CFZ (*P* = 0.7467) or TBI-166 (*P* = 0.9925) when comparing the MDR-TB and pre-XDR-TB subgroups (Fig. 1B).

### Epidemiological cutoff values and resistance rates

**Fig 2:**
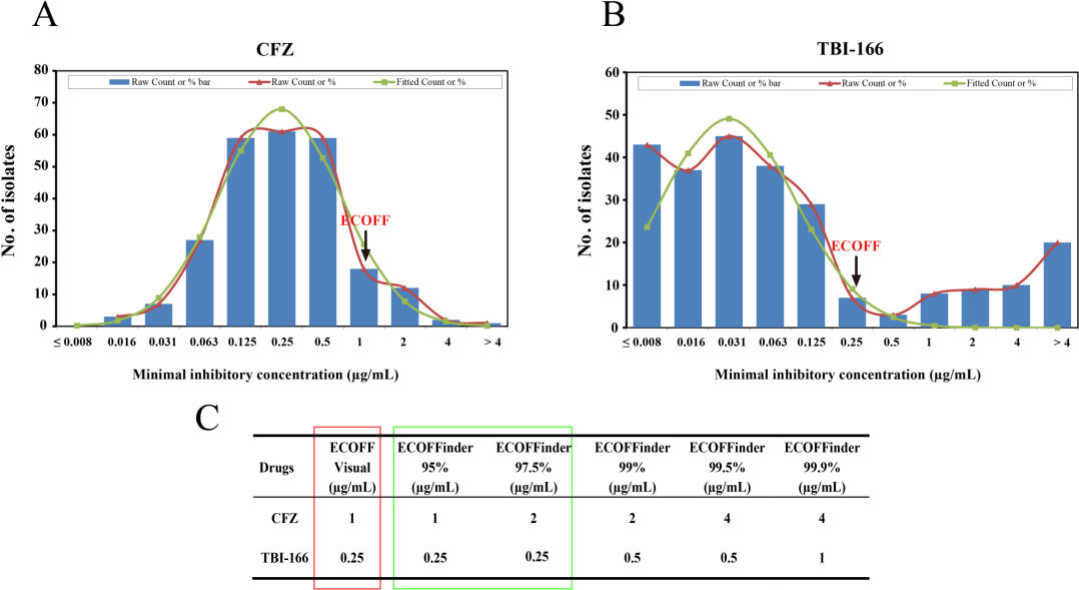
Nonlinear regression of MIC distribution of CFZ (**A**) and TBI-166 (**B**) to DR-B isolates. (**C**) The abscissa is MIC, the ordinate is the number corresponding to each MIC. The histogram is the original MIC distribution peak, the red curve is the original MIC distribution trend line, and the green curve is the fitting trend line. The arrows indicate the proposed CFZ and TBI-166 ECOFF values for DR-TB isolates. ECOFF, epidemiological cut-off values.

We further analyzed the tentative ECOFF values for CFZ and TBI-166. Based on visual inspection and statistical analysis using ECOFFinder software, tentative ECOFFs for MIC determination were set at 1.0 µg/mL for CFZ and 0.25 µg/mL for TBI-166 (Fig. 2). The CFZ ECOFF was consistent with resistance breakpoints used in previous *in vitro* studies. Using these *in vitro* ECOFFs, resistance to CFZ was detected in 6.1% (6/99) of MDR-TB and 6.0% (9/150) of pre-XDR-TB isolates, whereas resistance to TBI-166 was more prevalent at 19.2% (19/99) and 20.7% (31/150), respectively. A paired comparison using McNemar’s test confirmed that the resistance rates to TBI-166 were significantly higher than those to CFZ in both subgroups (*P* < 0.0001).

Notably, 28.0% (14/50) of the TBI-166-resistant isolates were cross-resistant to CFZ, while 72.0% (36/50) remained susceptible to CFZ (MICs: 0.063 µg/mL, *n* = 1; 0.125 µg/mL, *n* = 2; 0.25 µg/mL, *n* = 4; 0.5 µg/mL, *n* = 15; 1 µg/mL, *n* = 14). Resistance levels differed by drug: 80.0% (12/15) CFZ-resistant isolates exhibited low-level resistance (MIC ≤ 2 µg/mL), whereas 40.0% (20/50) of TBI-166-resistant isolates demonstrated high-level resistance (MIC > 4 µg/mL). This trend was more pronounced in pre-XDR-TB isolates, with 44.8% (13/29) of high-level TBI-166-resistant (MIC > 4 µg/mL) and 88.9% (8/9) of low-level CFZ-resistant isolates (MIC ≤ 2 µg/mL) being identified, highlighting a potential association with enhanced drug resistance profiles.

### Demographic/clinical factors associated with CFZ or TBI-166 resistance

Among the 249 clinical isolates, 162 (65.1%) were obtained from male patients, and 87 (34.9%) were from female patients. All isolates were derived from patients aged 16–77 years (mean ± SD, 43.1 years), including 41 from new cases and 208 from retreated cases (Table 2).

**TABLE 2:** Demographic/clinical risk factors associated with resistance to CFZ and TBI-166 in southwestern China^*a*^

Characteristic	No. (%) of isolates		OR (95% CI)	*P* value	No. (%) of isolates		OR (95% CI)	*P* value
	Resistance to CFZ (*n* = 15)	Sensitive to CFZ (*n* = 234)			Resistance to TBI-166 (*n* = 50)	Sensitive to TBI-166 (*n* = 199)		
Gender								
Men	6 (40%)	156 (66.7%)	1.0 (Ref)	0.04	30 (60.0%)	132 (66.3%)	1.0 (Ref)	0.41
Women	9 (60%)	78 (33.3%)	3.00 (1.03–8.73)		20 (40.0%)	67 (33.7%)	1.31 (0.69–2.49)	
Age group (yr)								
<25	3 (20%)	39 (16.7%)	1.46 (0.23–9.24)		7 (14.0%)	35 (17.6%)	0.80 (0.26–2.46)	
25–60	10 (66.7%)	157 (67.1%)	1.21 (0.26–5.75)	0.92	35 (70.0%)	132 (66.3%)	1.06 (0.45–2.51)	0.82
>60	2 (13.3%)	38 (16.2%)	1.0 (Ref)		8 (16.0%)	32 (16.1%)	1.0 (Ref)	
Treatment history								
New case	2 (13.3%)	39 (16.7%)	1.0 (Ref)	0.73	8 (16.0%)	33 (16.6%)	1.0 (Ref)	0.92
Retreatment	13 (86.7%)	195 (83.3%)	1.30 (0.28–5.99)		42 (84.0%)	166 (83.4%)	1.04 (0.45–2.43)	

^*a*^ OR, odds ratio; CI, confidence interval.

Univariate analyses were performed to assess associations between demographic/clinical factors (gender, age, treatment history) and resistance to CFZ or TBI-166. As summarized in Table 2, gender emerged as the only significant predictor of CFZ resistance (*P* = 0.041), with female patients exhibiting a threefold higher risk of resistance compared to males (odds ratio [OR] = 3.0, 95% confidence interval [CI]: 1.03–8.73). In contrast, no statistically significant associations were observed between TBI-166 resistance and gender, age, or treatment history (all *P* > 0.05).

### Mutations associated with CFZ and TBI-166 resistance

Of the 249 DR-TB isolates, 15 were CFZ-resistant, and 50 were TBI-166-resistant, with 93.3% (14/15) of CFZ-resistant isolates exhibiting cross-resistance to TBI-166. To elucidate the molecular basis of these resistance phenotypes, WGS was performed to characterize mutational profiles in *Rv0678*, *Rv1979c*, and *pepQ* (Table 3).

**TABLE 3:** Analysis of the *Rv0678* and *Rv1979c* mutations in CFZ/TBI-166-resistant clinical isolates

Genes	No. of isolates		Mutation	Amino acid	Type
	CFZ	TBI-166			
*Rv0678*	10	15			
	5	7	152CAG-CGG	Gln51Arg	Non-synonymous
	3	3	274T-TA	ins_A	Insert_frameshift
	1	1	471TAC-TAA	Y-stop	Non-synonymous
	1	1	374CTG-CGG	Leu125Arg	Non-synonymous
	0	1	23GAT-GCT	Asp8Ala	Non-synonymous
	0	1	157TCG-CCG	Ser53Pro	Non-synonymous
	0	1	-62/-21TT-		Delete
*Rv1979c*	1	6			
	1	1	−19C-T		Intergenic
	0	1	−70C-T		Intergenic
	0	1	−130T-G		Intergenic
	0	1	506GGC-GAC	Gly169Asp	Non-synonymous
	0	2	1276GTT-ATT	Val426Ile	Non-synonymous

Genetic analysis identified *Rv0678* mutations in 15 resistant isolates. Among these, 10 were co-resistant to both CFZ and TBI-166, with mutations including five isolates carrying a non-synonymous Pro51Ala substitution (CAG→CGG), three isolates harboring an Ins_A insertion at nucleotide position 274, one isolate containing a premature stop codon (TAC→TAA) at amino acid residue 157, and one isolate exhibiting a non-synonymous Leu125Arg (CTG→CGG) substitution. The remaining five *Rv0678*-mutated isolates were TBI-166 monoresistant. Two of these isolates harbored the Pro51Ala (CAG→CGG) mutation, while three carried mutations (Ser53Pro [TCG→CCG], Asp8Ala [GAT→GCT], and a TT deletion at positions −62/−21).

For the *Rv1979c* gene locus, six mutant isolates were identified, including one CFZ/TBI-166 cross-resistant isolate with an intergenic −19C→T mutation, and five TBI-166 monoresistant isolates (two with Val426Ile [GTT→ATT], one with Gly169Asp [GGC→GAC], and two with intergenic alterations at −70C→T and −130T→G, respectively). No clinically significant mutations were identified in *pepQ*, suggesting this gene does not contribute to the observed resistance phenotypes.

To explore genotype-phenotype associations, resistant isolates with *Rv0678* or *Rv1979c* mutations were further analyzed. For *Rv0678*, non-synonymous Gln51Arg substitutions were the most prevalent mutations in CFZ-resistant (50.0%, 5/10) and TBI-166-resistant (46.7%, 7/15) isolates. Insertion mutations (Ins_A) were the second most common, occurring in 30.0% (3/10) of CFZ-resistant and 20.0% (3/15) of TBI-166-resistant isolates. Phenotypically, *Rv0678-*mutated CFZ-resistant isolates primarily exhibited MICs of 2 µg/mL (90%, 9/10), twofold higher than the CFZ ECOFF (1.0 µg/mL), whereas TBI-166-resistant isolates with *Rv0678* mutations mostly showed MICs >4 µg/mL (66.7%, 10/15), eightfold higher than the TBI-166 ECOFF (0.25 µg/mL).

For *Rv1979c*, non-synonymous Val426Ile mutations were only detected in two TBI-166-resistant isolates, with other amino acid-altering mutations restricted to one isolate, indicating low prevalence and high dispersion. Corresponding MIC values were broadly distributed (0.5 to >4 µg/mL). While these mutations may contribute to high-level resistance, further functional validation is required to confirm their mechanistic role.

## DISCUSSION

The escalating global burden of MDR-TB and pre-XDR-TB remains a critical clinical challenge to public health, particularly in high-burden regions like southwest China (21). As a core second-line agent, CFZ and its optimized analog pyrifazimine (TBI-166, as China’s first anti-TB drug with independent intellectual property rights, with improved safety) show promise as a candidate for DR-TB management. This study directly characterized their *in vitro* activities against MDR/pre-XDR-TB isolates from southwestern China and explored underlying resistance mechanisms, providing insights into regional data gaps in riminophenazine efficacy and resistance.

Our MIC analyses revealed a striking concentration-dependent divergence between TBI-166 and CFZ (Table 1). At low concentrations (0.008–0.125 μg/mL), TBI-166 inhibited 77.1% (192/249) of DR-TB isolates, twice the rate of CFZ (38.6%, 96/249), confirming its superior potency at subtherapeutic levels, consistent with prior studies involving 28 MDR-TB isolates (11). However, at high concentrations (2 to >4 µg/mL), TBI-166 resistance (15.2%, 39/249) substantially exceeded that of CFZ (6.0%, 15/249). This aligns with Zhang et al.’s observation that high TBI-166 concentrations alter a higher NADPH/NADP^+^ ratio (22), potentially driven by structural modifications (e.g., C-3 pyridine ring adjustment) that affect lipid solubility and target binding. These data suggest TBI-166 may not substitute for CFZ in high-dose regimens, emphasizing the need for dose optimization guided by clinical pharmacokinetics in regions with distinct DR-TB prevalence, like southwest China.

**Fig 3:**
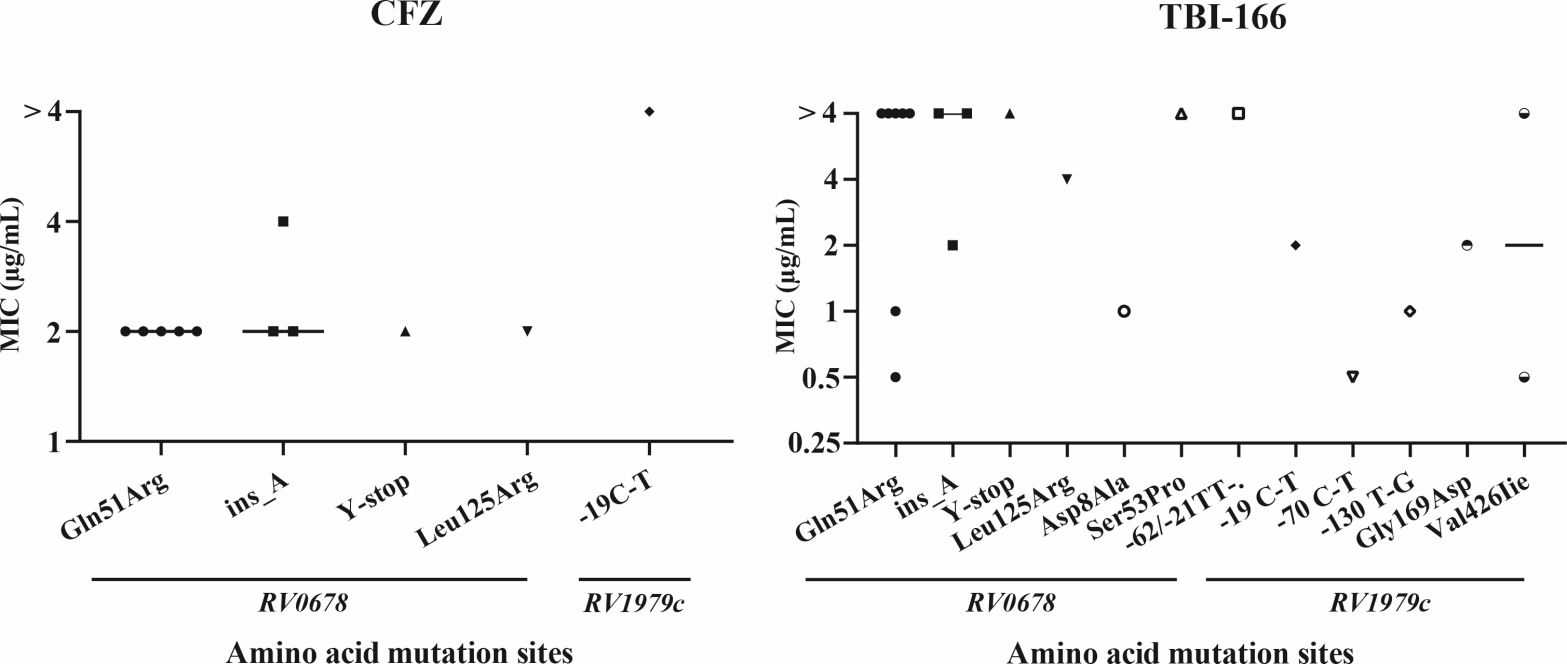
Distribution of MICs for CFZ- and TBI-166-resistant DR-TB isolates harboring amino acid mutations in *Rv0678* or *Rv1979c*. The x-axis indicates specific amino acid mutation sites in *Rv0678* or *Rv1979c* identified in CFZ-resistant or TBI-166-resistant isolates. The y-axis represents the corresponding MIC values (µg/mL) of CFZ or TBI-166 for isolates carrying each mutation. Each data point denotes an individual isolate, and each shape denotes a distinct amino acid mutation type (e.g., solid circles indicate Gln51Arg, solid squares denote ins_A, solid equilateral triangles represent Y-stop, and solid downward triangles signify Leu125Arg).

Mutant isolation frequencies further supported these trends. CFZ-resistant isolates were enriched at 2 μg/mL (9/11, Fig. 3), consistent with 80.0% of CFZ-resistant isolates exhibiting low-level resistance (MIC = 2 µg/mL), likely a consequence of widespread CFZ use in local MDR-TB treatment. In contrast, TBI-166-resistant isolates were most frequent at >4 µg/mL (11/21, Fig. 3), mirroring the high-level resistance (MIC > 4 µg/mL) in 40.0% of the TBI-166-resistant clinical isolates. We observed interannual fluctuations in CFZ and TBI-166 resistance rates (2017–2023), with TBI-166 resistance consistently higher (16.7%–33.3% vs 2.6%–25.0% for CFZ, Table S3) in years with detectable resistance (no resistance in 2021, likely due to *n* = 5). However, the uneven annual sample sizes and the small resistant isolate counts precluded robust statistical analysis of temporal trends, as is noted in the Limitations section. This pattern suggests TBI-166 may exert stronger selective pressure for high-level resistance at elevated concentrations. Given that many *in vitro* TBI-166-resistant isolates have high MICs, we speculate that high-level resistance could compromise its future clinical efficacy, emphasizing the need for proactive surveillance before clinical rollout, especially in regions with limited prior exposure to riminophenazine analogs.

Genetic analyses highlighted *Rv0678* as the primary resistance-associated gene, with Gln51Arg substitutions predominant in both CFZ-resistant (50.0%) and TBI-166-resistant (46.7%) isolates (Table 3). Classified as a Group 2 variant (clinically relevant phenotypic resistance marker) in the updated WHO mutation catalog for 2023 (23), Gln51Arg diverges from previously reported hotspots (nucleotides 193 and 466) and was rare (1/96, 1.04%) in a prior cohort (14). To investigate the potential phylogenetic clustering of resistant isolates, we constructed a phylogenetic tree for the 51 riminophenazine-resistant isolates using the SAM-TB platform, with results provided in Fig. S1A. Additionally, the isolates were distributed across four distinct lineages: 44 (86.3%) belonging to lineage 2.2.1, 2 (3.9%) to lineage 2.2.2, 3 (5.9%) to lineage 4.4.2, and 2 (3.9%) to lineage 4.5 (Fig. S1B), with no evidence of phylogenetic clustering (i.e., no shared recent common ancestor). This confirms that the observed resistance genotypes are not restricted to a single clonal lineage or source but reflect regionally prevalent lineages in southwestern China, highlighting the regional specificity of riminophenazine resistance mechanisms, which is of critical importance given the fact that these mechanisms were previously unknown in this region.

We also identified six novel *Rv0678* mutations, with plausible functional impacts: (i) Ins_A insertion (274T→TA) may disrupt the reading frame, altering protein structure; (ii) Y-stop (471TAC→TAA) is a premature stop codon, likely causing loss of function and disrupting *Rv0678*’s regulatory role; (iii) a -62/-21TT deletion could affect promoter activity; (iv) non-synonymous substitutions (Leu125Arg [CTG→CGG], Ser53Pro [TCG→CCG], Asp8Ala [GAT→GCT]) (Table 3) may impair target binding or protein stability. According to the WHO mutation catalog (23), Y-stop is classified as Group 3 and Ser53Pro as Group 2 (clinically relevant resistance marker), and the remaining four mutations are undocumented (potential novel loci). Phenotypically, all six mutations were associated with 2–8-fold elevated MICs relative to ECOFFs. While Ins_A was found in some susceptible isolates (Table S2), its enrichment in resistant isolates suggests it may act as a resistance determinant with incomplete penetrance, requiring functional validation.

By contrast, mutations in *Rv1979c* were sparse and diverse (−19C→T, −130T→G, −70C→T, Val426Ile [GTT→ATT] and Gly169Asp [GGC→GAC]; Table 3), distinct from previously reported resistance-associated variants (Val52Gly, Val351Ala) (14, 24) and mostly classified as Group 3/4 (uncertain or non-resistance markers) by the 2023 WHO mutation catalog (23) Specifically, −19C→T and −130T→G are Group 3 variants (uncertain from available evidence), −70C→T and Val426Ile are Group 4 variants (not markers of resistance) and the undocumented Gly169Asp is a novel locus. The Val426Ile mutation was also detected in TBI-166-susceptible isolates and paired with broad MIC ranges (0.5–4 µg/mL). This suggests *Rv1979c* plays a secondary role in riminophenazine resistance in this cohort from southwestern China. The absence of clinically significant *pepQ* mutations in resistant isolates contrasts with reports of loss-of-function variants driving resistance (15), further supporting geographically distinct resistance pathways. For exploratory TBI-166-associated genes (*Rv2621c*, *Rv2622*, *Rv3066*, and *Rv3418c*) (20), we detected an Ala110Val non-synonymous mutation in *Rv2621c* (Tables S1 and S2) in all TBI-166-resistant isolates (but also susceptible isolates, indicating lineage-specific variation) and *Rv2622* mutations (Ala8Val, Val207Ala) in two TBI-166-resistant isolates (Table S1). These findings do not support a direct role in riminophenazine resistance, aligning with the CRyPTIC Consortium’s observation that no single gene fully explains CFZ resistance (25), pointing to multifactorial mechanisms that may vary by region.

We included demographic/clinical factors (gender, age, treatment history) based on their established role in modulating treatment adherence, immune responses, and drug exposure, key drivers of DR-TB resistance (26, 27). Gender was the only significant predictor of CFZ resistance (*P* = 0.041; OR = 3.0, 95% CI:1.03–8.73), but this preliminary finding requires further consideration, as it may be due to the limited power of univariate analysis (*n* = 15 CFZ-resistant isolates). No associations were observed for TBI-166, likely due to the long-term clinical use of CFZ inducing gender-specific selective pressure as opposed to TBI-166 resistance driven by CFZ cross-resistance.

This study has several limitations. Firstly, although our cohort reflects the high TB burden in southwestern China, validating our ECOFFs across broader geographic regions, including other global DR-TB hotspots, such as South Asia and sub-Saharan Africa, would make them more generalizable. Secondly, isolates were selected from a biobanked collection based on cryopreservation viability (not random sampling), which introduces potential selection bias. Additionally, the uneven annual sample sizes (e.g., *n* = 5 in 2021 vs *n* = 87 in 2018) preclude robust statistical analysis of temporal resistance trends, and both factors limit generalizability to the broader regional DR-TB population. Thirdly, the relatively small number of resistant isolates (particularly 15 CFZ-resistant isolates) not only hinders the establishment of robust genotype-phenotype correlations (requiring larger-scale studies integrating WGS and functional assays to confirm novel mutations like Rv0678 Ins_A) but also limits the power of univariate risk factor analysis, with the preliminary association between female gender and CFZ resistance requiring validation in larger cohorts. Fourthly, the *in vitro* ECOFFs for CFZ and TBI-166 should be integrated with pharmacokinetic/pharmacodynamic data and clinical outcomes (28), which are needed to refine susceptibility thresholds for the clinical use of TBI-166. Finally, *in vitro* findings require *in vivo* validation (e.g., murine models, clinical trials) to confirm clinical relevance, particularly with regard to the concentration-dependent efficacy of TBI-166.

### Conclusions

Our study demonstrates distinct *in vitro* activity profiles of TBI-166 and CFZ against MDR/pre-XDR-TB isolates from southwest China, with TBI-166 exhibiting superior low-concentration potency but higher high-concentration resistance. Novel *Rv0678* mutations, alongside sparse *Rv1979c* variants and the absence of *pepQ* mutations, reveal region-specific riminophenazine resistance mechanisms. These findings inform regional therapeutic optimization, support the need for localized resistance surveillance, and contribute to global efforts to understand the diverse geographical phenotypes of DR-TB.

## Data Availability

The raw genomic sequencing data reported in this study have been deposited in the NCBI Sequence Read Archive (SRA) under BioProject accession number PRJNA1423053. Variant call tables for these MTB isolates are available in the ScienceDB repository at https://cstr.cn/31253.11.sciencedb.28573.

## SUPPLEMENTAL MATERIAL

Supplemental figures and tables spectrum.02664-25-s0001.docx Fig. S1 and Tables S1 to S3.

Fig. S1 spectrum.02664-25-s0002.tiff Phylogeny, sublineage structure, and drug resistance profiles of the 51 riminophenazine-resistant (CFZ + TBI-166) clinical isolates.

ASM does not own the copyrights to Supplemental Material that may be linked to, or accessed through, an article. The authors have granted ASM a non-exclusive, world-wide license to publish the Supplemental Material files. Please contact the corresponding author directly for reuse.
