# Supplementary material for: Comparative in vitro antimicrobial susceptibility profiles of clofazimine and pyrifazimine against clinical isolates of Mycobacterium tuberculosis in southwest China
Source: Microbiol Spectr. 2026 Jul 6;14(8):e02664-25. doi: 10.1128/spectrum.02664-25 (PMC13435795; doi:10.1128/spectrum.02664-25)
Supplement: Supplementary file 1 [file spectrum.02664-25-s0001.docx]

Table S1. The *Rv2621c* and *Rv2622* mutations for the 51 riminophenazine-resistant (CFZ + TBI-166) clinical isolates

| **Genes** | **No. of isolates** | | **Mutation** | **Amino acid** | **Type** |
| --- | --- | --- | --- | --- | --- |
|  | **CFZ** | **TBI-166** |  |  |  |
| ***Rv2621c*** |  |  |  |  |  |
|  | 0 | 1 | 199CGC-AGC | Arg67Ser | Nonsynonymous |
|  | 15 | 50 | 329GCC-GTC | Ala110Val | Nonsynonymous |
| ***Rv2622*** |  |  |  |  |  |
|  | 0 | 1 | 23GCC-GTC | Ala8Val | Nonsynonymous |
|  | 0 | 1 | 620GTC-GCC | Val207Ala | Nonsynonymous |

Table S2. The *Rv0678*, *Rv1979c, Rv2535c* (*pepQ*), *Rv2621c,* *Rv2622* and *Rv3418c* mutations for the riminophenazine-sensitive clinical isolates

| **Genes** | **No. of isolates** | | **Mutation** | **Amino acid** | **Type** |
| --- | --- | --- | --- | --- | --- |
|  | **CFZ** | **TBI-166** |  |  |  |
| ***Rv0678*** |  |  |  |  |  |
|  | 1 | 1 | 54A-AT | ins_T | Insert_frameshift |
|  | 1 | 1 | 138T-TG | ins_G | Insert_frameshift |
|  | 1 | 1 | 274T-TA | ins_A | Insert_frameshift |
|  | 1 | 1 | 155TCC-TAC | Ser52Tyr | Nonsynonymous |
|  | 0 | 1 | 254GTC-GGC | Val85Gly | Nonsynonymous |
|  | 0 | 1 | 311GAG-GGG | Glu104Gly | Nonsynonymous |
|  | 0 | 1 | 421GAT-CAT | Asp141His | Nonsynonymous |
| ***Rv1979c*** |  |  |  |  |  |
|  | 1 | 1 | 506GGC-GAC | Gly169Asp | Nonsynonymous |
|  | 7 | 7 | 1276GTT-ATT | Val426Iie | Nonsynonymous |
| ***pepQ*** |  |  |  |  |  |
|  | 1 | 1 | 648GGC-GAC | Asp216Glu | Nonsynonymous |
|  | 2 | 2 | 806GTT-ATT | Glu269Gly | Nonsynonymous |
| ***Rv2621c*** |  |  |  |  |  |
|  | 234 | 199 | 329G-A | Ala110Val | Nonsynonymous |
| ***Rv2622*** |  |  |  |  |  |
|  | 1 | 1 | 23GCC-GTC | Ala8Val | Nonsynonymous |
|  | 1 | 1 | 679GCT-AGT | Glu227Ser | Nonsynonymous |
| ***Rv3418c*** |  |  |  |  |  |
|  | 1 | 1 | -219GGC-GAC | Asp216Glu | Intergenic |

Table S3. Annual resistance rates of clinical drug-resistant tuberculosis (DR-TB) isolates to CFZ and TBI-166 in Chongqing, China, 2017–2023. Data are presented as the number of resistant isolates/total isolates (resistance rate, %). Annual resistance rates of clinical DR-TB isolates to CFZ and TBI-166 during 2017 –2023

| **Year** | **Total isolates** | **CFZ - resistant isolates**  **(n / rate)** | **TBI-166 – resistant isolates**  **(n / rate)** |
| --- | --- | --- | --- |
| 2017 | 18 | 2/11.1% | 6/33.3% |
| 2018 | 87 | 5/5.7% | 18/20.7% |
| 2020 | 16 | 4/25% | 3/18.8% |
| 2021 | 5 | 0/0% | 0/0% |
| 2022 | 39 | 1/2.6% | 9/23.1% |
| 2023 | 84 | 3/3.6% | 14/16.7% |

Figure S1. Phylogeny, sublineage structure, and drug resistance profiles of the 51 riminophenazine-resistant (CFZ + TBI-166) clinical isolates. (A) Maximum‑likelihood phylogenetic tree was constructed from whole‑genome sequences, illustrating the genetic relatedness among the isolates. (B) Tabulated resistance phenotypes to CFZ and TBI‑166 alongside sublineage classification. Resistance and susceptibility are denoted as “1” and “0”, respectively. The collection predominantly belongs to sublineage 2.2.1 (n = 44) and also includes isolates from sublineages 2.2.2 (n = 2), 4.4.2 (n = 3), and 4.5 (n = 2).
